# Protective Effects of *Parkia biglobosa* Protein Isolate on Streptozotocin-Induced Hepatic Damage and Oxidative Stress in Diabetic Male Rats

**DOI:** 10.3390/molecules22101654

**Published:** 2017-10-02

**Authors:** Bolajoko Idiat Ogunyinka, Babatunji Emmanuel Oyinloye, Foluso Oluwagbemiga Osunsanmi, Andrew Rowland Opoku, Abidemi Paul Kappo

**Affiliations:** 1 Biotechnology and Structural Biology (BSB) Group, Department of Biochemistry and Microbiology, University of Zululand, KwaDlangezwa 3886, South Africa; bolajokotimi@gmail.com (B.I.O.); tunji4reele@yahoo.com (B.E.O.); alafin21@yahoo.com (F.O.O.); OpokuA@unizulu.ac.za (A.R.O.); 2 Department of Biochemistry, College of Sciences, Afe Babalola University, PMB 5454, Ado-Ekiti 360001, Nigeria

**Keywords:** hepatic antioxidant, histology, HPLC, oxidative stress, *Parkia biglobosa*, STZ-induced diabetes

## Abstract

This study sought to investigate the possible protective role of *Parkia biglobosa* seed protein isolate (PBPi) against streptozotocin-induced hepatic damage and oxidative stress in diabetic male rats. Prior to animal experiments, a HPLC fingerprint of PBPi was recorded. Diabetes was induced in rats by a single intraperitoneal injection of streptozotocin (STZ; 60 mg/kg body weight). Diabetic rats were orally treated daily with PBPi (200 or 400 mg/kg body weight) or insulin (5 U/kg, i.p.) for 28 days. The degree of protection was evaluated using biochemical parameters such as malondialdehyde (MDA) levels, serum transaminases (ALT and AST), total protein, total glutathione (Total GSH), glutathione-S-transferase (GST), superoxide dismutase (SOD), catalase (CAT), and interleukin-6 (IL-6) activities. Histology of liver sections was also performed. The HPLC fingerprint of PBPi revealed eleven distinct peaks; PBPi at tested doses significantly attenuates STZ-induced elevated levels of serum IL-6, ALT and AST; and hepatic TBARS levels. Hepatic antioxidants (Total GSH, GST, SOD, CAT) as well as total protein were markedly restored in a dose-dependent manner. Histopathological results strongly support the protective role of PBPi. These results suggest PBPi could confer protection by ameliorating hepatic damage and oxidative stress caused by STZ in animal model possibly via its anti-inflammatory and antioxidant properties.

## 1. Introduction

The global day-to-day prevalence of diabetes mellitus, a group of metabolic disorders, has increased at an alarming rate. It has recently been estimated that by the year 2030 there is the possibility for about 366 million people to be diabetic worldwide [1,2]. Significant increase in morbidity and mortality rates in diabetics has been associated with microvascular (retinopathy, neuropathy, and nephropathy) and macrovascular (heart attack, stroke and peripheral vascular disease) complications [3,4,5,6]. At the moment there is no total cure for diabetes, therefore this complex multifactorial disease requires a lifelong management. Globally, prevention or reversal of diabetes using anti-diabetic drugs without any side effect is still a key challenge [7,8]. In the etiology of diabetes, it is generally accepted that diabetes is characterized by uncontrolled hyperglycaemia that is developed due to ineffective insulin secretion, inadequate insulin or both as well as disturbances of carbohydrate, protein and fat metabolism resulting in free radicals (especially reactive oxygen species; ROS) induced oxidative stress and oxidative damage. This process has been suggested as the mechanism underlying diabetes and its associated complications [9,10]. Under normal physiological circumstances, insulin regulates carbohydrate, lipid and amino acid metabolism as well as mRNA transcription and translation. Insulin resistance in most cases is believed to be manifested at the cellular level through post-receptor defects in insulin signalling. The possible mechanisms may include down-regulation, deficiencies or genetic polymorphisms of tyrosine phosphorylation of the insulin receptor, IRS proteins or PIP-3 kinase, or may involve abnormalities of GLUT 4 function [11]. 

In addition, non-enzymatic protein glycation, impaired antioxidant enzymes status and production of peroxides, which may contribute to severe liver damage and disorder, have also been implicated as a possible source of oxidative stress. Increasing evidence suggests that oxidative stress plays an important role in the pathogenesis and progression of diabetes mellitus [7]. Taken together, excessive hyperglycaemia, oxidative stress and hepatic fat accumulation play a vital role in the development of hepatocellular injury in diabetics [12]. Added to these, a direct relationship has been established between increased cytokines, such as tumour necrosis factor-α (TNF-α), interleukin 6 (IL-6) and interleukin 10 (IL-10) and excessive hyperglycaemia in both types 1 and 2 diabetes [13,14]. IL-6 has emerged as an important systemic alarm signal, which is usually elevated in response to almost every kind of damage or tissue injury [15]. Under hyperglycaemic condition, the elevated glucose and ROS levels are pro-inflammatory and may increase the level of IL-6. This evidence confirms a role for cytokines in types 1 and 2 diabetes [16,17]. Several pharmacological applications of plant-derived foods (nutraceuticals) towards improving the immune defence and reducing risks associated with diabetes have been reported in literature [7]. Detailed evaluation of some of these plant-derived foods may serve as potential source of novel therapy in the prevention or reversal as well as in the management of diabetes and its associated complications. *P. biglobosa* has been identified as one of such plants possessing hepatoprotective, anti-inflammatory, antioxidative as well as hypolipidaemic and antimicrobial properties [7]. *P. biglobosa* is an essential household spice usually consumed in Nigeria and many other West African countries in the preparation of various foods as well as a seasoning agent in soups [7,18,19]. This study was therefore designed to evaluate the protective activities of protein isolate from *Parkia biglobosa* seeds (PBPi) on streptozotocin-induced hepatic damage and oxidative stress in diabetic male rats. 

## 2. Results

### 2.1. HPLC Analysis

**Figure 1:**
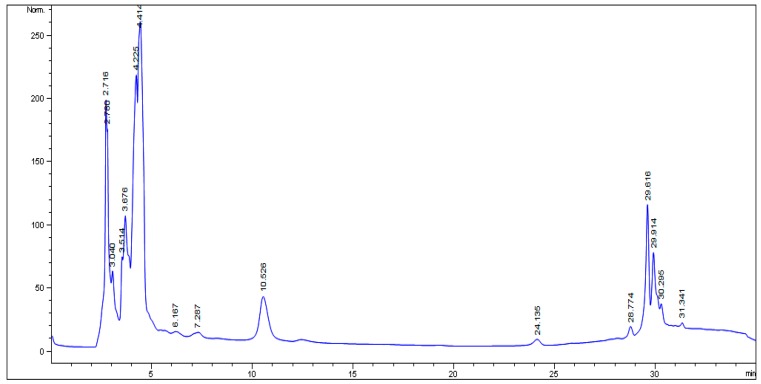
HPLC fingerprint of *Parkia biglobosa* protein isolate.

The HPLC fingerprint (Figure 1) of *Parkia biglobosa* protein isolate obtained revealed eleven (11) distinct peaks at the following retention times (minutes): 2.716, 2.780, 3.040, 3.514, 3.676, 4.225, 4.414, 10.526, 29.616, 29.914 and 30.295. 

Five (5) minor peaks at the following retention times (minutes): 6.167, 7.287, 24.135, 28.774 and 31.341 were also observed.

### 2.2. Effect of PBPi on Serum Liver Function

**Figure 2:**
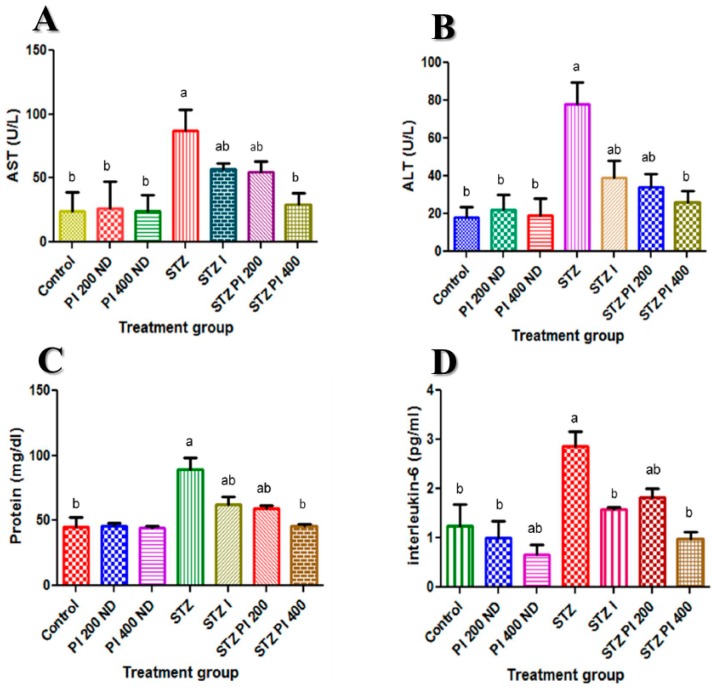
Effect of PBPi on serum liver function and serum interleukin-6 (IL-6). (**A**) AST (U/L), (**B**) ALT (U/L), (**C**) Protein (mg/dL), (**D**) Interleukin-6 (pg/mL). Data are presented as mean ± S.D. (n = 10). Mean differences are significant (*p* < 0.05) when compared with: ^a^ control group, ^b^ STZ only.

Presented in Figure 2A–C is the effect of PBPi on serum liver function indices (AST and ALT) as well as serum total protein respectively. Serum activities of AST and ALT were significantly elevated with a concomitant decrease in the serum total protein level in STZ-induced diabetic rats when compared with the control. Treatment with PBPi attenuated these changes in a dose-dependent manner.

### 2.3. Effect of PBPi on Serum Interleukin-6 (IL-6)

In diabetic rats, there was a significant increase in the serum interleukin-6 level when compared with the control. When PBPi was administered to the diabetic rats, there was a significant down-regulation in the production of interleukin-6; this was exhibited in a dose-dependent manner, which was comparable to that of insulin (Figure 2D).

### 2.4. Effect of PBPi on Assessment of Oxidative Stress and Antioxidants

**Figure 3:**
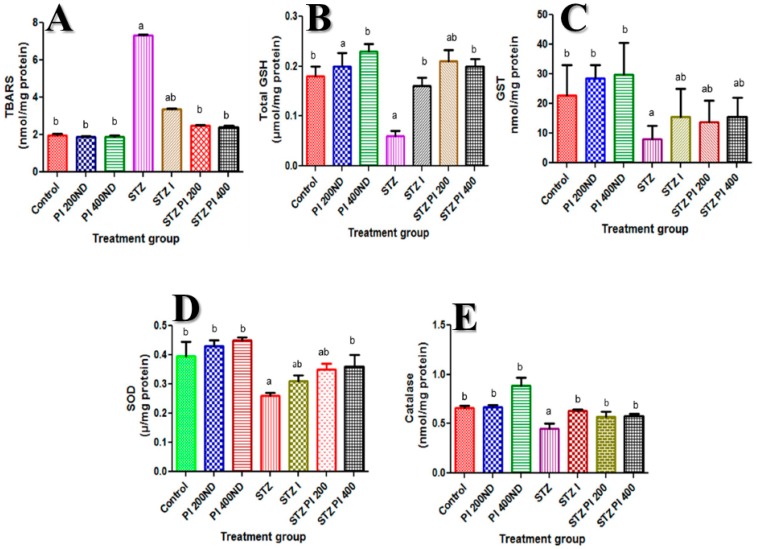
Effect of PBPi on assessment of oxidative stress and antioxidants. (**A**) TBARS (nmol/mg protein), (**B**) Total GSH (μmol/mg protein), (**C**) GST (nmol/mg protein), (**D**) SOD (μ/mg protein), (**E**) CAT (nmol/mg protein). Data are presented as mean ± S.D. (n = 10). Mean differences are significant (*p* < 0.05) when compared with: ^a^ control group, ^b^ STZ only. Thiobarbituric acid reactive substances (TBARS), total reduced glutathione (Total GSH), glutathione-S-transferase (GST), superoxide dismutase (SOD), catalase (CAT).

STZ caused a substantial increase in the hepatic TBARS content, which was associated with a concomitant significant decline in hepatic total GSH content as well as GST, SOD and CAT activities compared to the control. These changes were ameliorated when PBPi was administered for 28 days (Figure 3). 

### 2.5. Effect of PBPi on Liver Histopathological Examination

**Figure 4:**
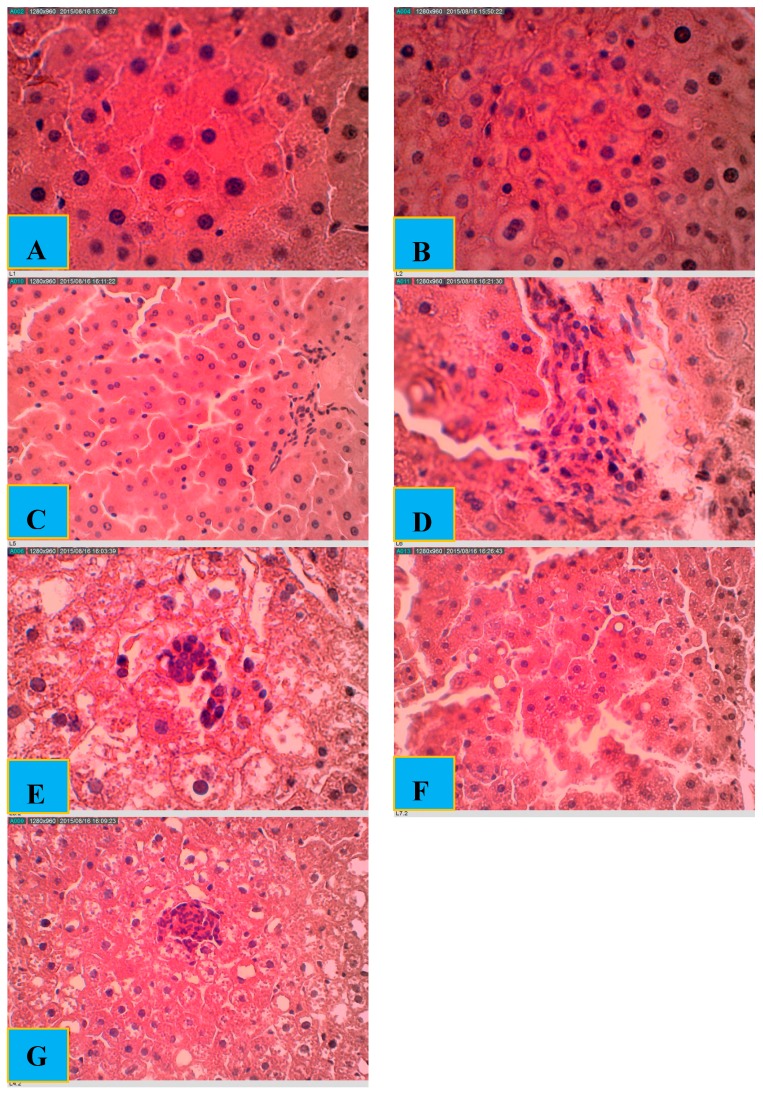
Histological examination of rat livers stained with hematoxylin and eosin (H&E). (**A**) Control: showing liver tissue with normal architecture; (**B**) PI 200ND: showing liver tissue with normal architecture; (**C**) PI 400ND: showing liver tissue with normal architecture; (**D**) STZ: showing expanded portal tracts by a severe lymphocytic infiltrate associated with necrotic hepatocytes focally; (**E**) STZ I: showing liver tissue with a mild lymphocytic infiltrate present within the portal tracts (**F**) STZ PI 200: showing the presence of very mild lymphocytic infiltrate within the portal tracts and the lobules, with mild focal necro-inflammatory changes; (**G**) STZ PI 400: showing regenerative changes within the hepatocytes even though there are very mild necro-inflammatory foci. (Scale bar = 310 μm).

The histological investigation of the liver tissue (Figure 4) showed normal architecture in the case of control, PI 200ND and PI 400ND groups. The portal tracts are within normal limits with no identified inflammatory cell infiltrate. The hepatocytes also showed normal histology, with no steatosis, inflammation, necrosis or cholestasis. In case of the STZ group, histopathological examination of the liver sections demonstrates that the portal tracts were expanded by a severe lymphocytic infiltrate associated with necrotic hepatocytes focally (these features are expressed as lobular and portal tract chronic inflammation and focal hepatocyte necrosis). In the STZ I group, sections showed liver tissue with a mild lymphocytic infiltrate present within the portal tracts. The inflammatory process is also observed within the hepatocytes with mild focal necro-inflammatory damage. Very mild lymphocytic infiltrate was observed within the portal tracts and the lobules of the STZ PI 200 group with mild focal necro-inflammatory changes. In addition, there was a very mild macrovesicular steatosis. In the STZ PI 400 group, sections demonstrated liver tissue with normal architecture. Lymphocytic infiltrate was identified within the portal tracts with no evidence of interface hepatitis. Regenerative changes were seen within the hepatocytes even though there are very mild necro-inflammatory foci. 

## 3. Discussion and Conclusions

Increasing experimental evidence has demonstrated that STZ-induced toxicity in animal models is characterized by insulin deficiency and hyperglycaemia [20,21]. When this condition is not properly managed, it results in extremely high intracellular ROS production, which is capable of interfering with structural macromolecules that in turn facilitates functional abnormalities as well as histological alterations [5,22,23]. 

Serum transaminases (ALT and AST) are important indicators of hepatocellular injury. These enzymes are usually abundant in the liver where they play a central role in amino acid metabolism. STZ-toxicity has been characterized by changes in the permeability of the liver membrane and cellular leakage of ALT and AST from the hepatocytes into the blood stream. In this study, the activities of these enzymes (ALT and AST) in serum of STZ-induced diabetic rats were markedly elevated. This suggests that there is a certain degree of damage to the liver. Similar reports appear in literature [24,25,26]. Treatment with PBPi at both doses (200 and 400 mg/kg body weight) considerably decreased the elevated levels of ALT and AST in serum of STZ-induced diabetic rats. Apparently, PBPi plays some protective roles in STZ-induced diabetic rats. 

Importantly, lapses in amino acid/protein metabolism as a result of deficiency in insulin secretion and/or inadequate insulin in STZ-induced diabetes are more critical factors than hyperglycaemia associated with some diabetic complications [27]. Documented experimental evidence demonstrates that STZ-diabetic rat model shows numerous alterations in amino acid metabolism. This has been linked to increased muscle proteolysis, reduced protein synthesis, an energy-dependent process in the liver, and stimulated hepatic gluconeogenesis utilizing gluconeogenic amino acids [27,28]. In this study, a significant reduction in the total serum protein levels was observed in STZ-induced diabetic rats. The reduction was significantly reversed after the treatment with PBPi in a dose-dependent manner. This is in agreement with similar studies reported in literature [29,30,31]. This could be attributed to the nutritional quality (high protein content; broad spectrum of essential amino acids) of PBPi. 

The literature has established that elevated levels of inflammatory mediators such as pro-inflammatory cytokines (IL-6 and IL-1β) in diabetes and its associated complications is as a result of hyperglycaemia and these mediators have been considered to be the link between inflammation and insulin resistance [14,16]. In agreement with previous similar studies [32,33,34], treatment with PBPi significantly inhibited the elevated level of IL-6 in the diabetic rats at the doses tested. The observed effect suggests that PBPi possess immuno-modulatory and anti-inflammatory properties.

It is believed that in STZ-diabetic conditions, hyperglycaemia produces ROS that exceeds endogenous antioxidant capacity; these ROS may react with polyunsaturated fatty acids of cell membranes thereby causing lipid peroxidation and membrane damage [35,36]. The result of the present study revealed that TBARS level in the liver of STZ-induced diabetic rats was markedly elevated when compared with the control. The concurrent treatment of diabetic rats with PBPi (200 and 400 mg/kg b.wt) showed a diminished level of TBARS. Our findings are in agreement with earlier experimental reports [37,38,39]. The observed effect suggests that there was an amelioration of oxidative stress in diabetic rats, which may be due to the free radical scavenging activity of PBPi.

Besides the elevation witnessed in the TBARS levels in the STZ-induced diabetic rats, our results demonstrated that there was a concomitant decline in the hepatic antioxidant capacity notably total GSH, GST, SOD and CAT. Hyperglycaemia is known to inactivate the activities of antioxidative enzymes in diabetic animal models, which may involve non-enzymatic glycosylation [40]. Antioxidant enzymes as well as non-enzymatic antioxidants are first line of defence against ROS-induced oxidative damage in a living organism [22]. 

Added to this, SOD and CAT play an essential role in attenuating cellular stress by maintaining physiological levels of oxygen and hydrogen peroxide. SOD scavenges the superoxide radical by converting it to hydrogen peroxide and molecular oxygen, while CAT brings about the reduction of hydrogen peroxides and protects higher tissues from the highly reactive hydroxyl radicals [35,41]. Interestingly, administration of PBPi to STZ-induced diabetic rats considerably revert the hepatic antioxidant status in a dose-dependent manner near normal when compared with the control group. 

The HPLC fingerprint obtained for PBPi revealed 11 distinct peaks and five minor peaks, which should prove helpful in providing new leads for future identification of the bioactive components that are, individually and/or synergistically, responsible for the protective effect of PBPi in this study. Basically, the bioactive components identified in PBPi are believed to be insulin-like protein with insulin-releasing activity. The protective effect of PBPi was furthermore strongly supported by the results of the histopathological examination (Figure 4). Taken in all, the implication of the results obtained in the present study shows that PBPi could improve tissue’s insulin sensitivity, which may lead to insulin restoration and thereby improve the imbalance in carbohydrate, lipid and amino acid/protein metabolism experienced during diabetes. The most likely molecular mechanism(s) by which PBPi elicit its pharmacological effect in STZ-induced diabetic rats in this study could be associated with the modulation of metabolic abnormalities in the hyperglycemic state via the inhibition of reactive oxygen species and suppression of the production of pro-inflammatory cytokines as well as the ability to attenuate the extent of lipid peroxidation due to its strong antioxidant potential.

In summary, it is apparent from the data obtained in this study that PBPi possess anti-inflammatory and antioxidant properties capable of protecting against STZ-induced hepatic injury and oxidative stress in diabetic animal models. Therefore, in the ongoing search for novel therapy for diabetes management, PBPi is a promising candidate.

## 4 Materials and Methods

### 4.1. Chemicals

All the chemicals used in this study were of analytical grade and were purchased from Sigma Chemical Co. (St. Louis, MO, USA), Merck (Modderfontein, South Africa) and ScienCell Research Laboratories (Carlsbad, CA, USA).

### 4.2. Plant Material and Preparation of the Protein Isolate

After obtaining import permit (P0060156) from the Department of Agriculture, Forestry and Fisheries (DAFF; Pretoria, South Africa) raw and fermented *Parkia biglobosa* seeds used in this study were purchased from a local market in Ijebu-Ode (Ogun State, Nigeria). The seeds were identified and authenticated by the Chief Botanist of the Department of Botany, University of Zululand and a voucher specimen (B07) was deposited in the University Herbarium. 

Prior to separation of protein isolate, the fermented seeds were oven-dried at 50 °C; thereafter the dried seeds were ground into uniform powder using an electric blender. One kilogram of uniform powdered seeds was defatted with 2000 mL of n-hexane to obtain the defatted extract. The defatted extract was air-dried and then extracted with butanol (1:10 *w*/*v*) to remove possible anti-nutrients. Protein isolate was obtained from the defatted extract using the method described by Nkosi and colleagues [20]. Briefly, the dry defatted extract (1 kg) was re-suspended in distilled water (2000 mL, adjusted to pH 10 with 10 M NaOH). The resultant suspension was filtered to remove debris and the filtrate adjusted to pH 5, followed by centrifugation at 5000 rpm for 15 min at 4 °C. The supernatant was discarded while the pellet containing the protein isolate was retained and freeze-dried to yield a brown extract. The lyophilized extract was kept dry until needed. 

### 4.3. Induction of Diabetes in Experimental Animals

Seventy healthy, male, Sprague-Dowley rats (250–290 g; averaging 12 weeks old) were procured from the Animal House of the Department of Biochemistry and Microbiology, University of Zululand. The animals were acclimatized for 7 days prior to the commencement of the study; they were maintained at standard conditions of temperature and relative humidity, with a 12-h light/dark cycle. The animals were provided with standard rat pellets and water ad libitum. All animal experimental procedures were performed following the guideline for care and supervision of experimental animals and were in agreement with the Institutional Animal Ethics Committee clearance certificate (UZREC 171110-030-RA level 02 Dept 2014/74) issued by the University of Zululand Research Ethics Committee. 

Following an overnight fasting, diabetes was induced in the selected rats by a single intraperitoneal injection of freshly prepared STZ (Sigma-Aldrich Co., St. Louis, MO, USA) at a dose of 60 mg/kg body weight; dissolved in 0.1 M, pH 4.5 ice-cold citrate buffer [42]. Diabetes was confirmed in the rats 72 h after STZ administration by measuring the fasting blood glucose (FBG) levels. Rats with FBG level above 300 mg/dL were considered diabetic and selected for the study. Treatment commenced on the fourth day and continued for a period of twenty-eight days. 

### 4.4. Experimental Design and Biochemical Analysis

Animals were divided into seven groups of ten animals each and treated as follows: Group 1 (control) was given citrate buffer only. Group 2 (PI 200ND), non-diabetic rat, was given citrate buffer and protein isolate (200 mg/kg body weight). Group 3 (PI 400ND), non-diabetic rat, was given citrate buffer and protein isolate (400 mg/kg body weight). STZ-induced diabetic rats were divided in four groups (Groups 4–7). Group 4 (STZ), diabetic control. Group 5 (STZ I), positive control, was given insulin (5 U/kg, i.p.). Group 6 (STZ PI 200) diabetic rats that received protein isolate (200 mg/kg body weight). Group 7 (STZ PI 400) diabetic rats that received protein isolate (400 mg/kg body weight). Treatments were given orally for 28 days. The dose selection takes into account the future use of PBPi in humans with careful consideration of the pharmacokinetics and pharmacodynamics differences among species.

At the end of the 28 days of treatment, the rats were fasted overnight and then sacrificed under anaesthesia; blood samples were obtained by cardiac puncture in plain tubes without anticoagulants, left for 1 h to coagulate then centrifuged at 3000 rpm for 15 min at 4 °C to obtain serum samples. The liver was collected, washed in saline, blotted dry and weighed. Portions of the rats’ liver were homogenized in 56 mM Tris-HCl buffer (pH 7.4) containing 1.15% KCl, and then centrifuged at 10,000× *g* for 15 min to obtain the supernatants that were stored at −80 °C until needed for analysis. The other liver portions were preserved in 10% formalin and used for histological assessment of the liver. Histological studies were carried out at the Vet Diagnostix Laboratories (Pietermaritzburg, South Africa) by a qualified pathologist having no prior knowledge of the group the rat livers belonged. This method allowed for unbiased description of histological lesions, which were, present or absent in the samples. The liver tissues were stained with haematoxylin and eosin (H&E).

The extent of lipid peroxidation in the liver homogenate was determined spectrophotometrically by measuring the formation of malondialdehyde (MDA) according to the method described by Varshney and Kale [43], and this was expressed in terms of malondialdehyde (MDA) levels, which is the end product of the reaction. Serum activity of ALT, AST and the concentrations of total protein were measured by using commercial kits (Randox Laboratories Ltd., Crumlin, UK) specific for each test; the concentrations of the serum interleukin-6 (IL-6) was estimated using the enzyme-linked immunosorbent assay kit (Thermo Scientific, Waltham, MA, USA) while the hepatic level of total glutathione (Total GSH) as well as the activities glutathione-S-transferases (GST), superoxide dismutase (SOD) and catalase (CAT), were determined by using the corresponding assay kits (ScienCell Research Laboratories) according to the manufacturer’s instructions.

### 4.5. HPLC Analysis

The HPLC analysis of *Parkia biglobosa* protein isolate (PBPi) was carried out at the University of the Western Cape, Cape Town, South Africa. Chromatographic system: Beckman (Coulter GmbH, Krefeld, Germany) HPLC system consisting of a double pump Programmable Solvent Module model 126; Diode Array detector module model 160; Samsung computer 386 with System Gold (V601) management software provided by Beckman; Column: C_18_ Bondapak 5 µm and dimensions 250 × 4.6 mm^2^ was used. Chromatographic conditions: Mobile phase, solvent A: 1% acetic acid; solvent B: methanol, Mode: gradient flow rate, 1 min/min; injection volume, 10 µL; detector, UV at 350 nm. The HPLC operating conditions were programmed to give the following: 0 min, solvent B: 20%; 5 min, solvent B: 40%; 15 min, solvent B: 60%; 20 min, solvent B: 80% and 27 min. The run rate was 30 min [44].

### 4.6. Statistical Analysis

All data are presented as the mean ± standard deviation. The data were analyzed by one-way ANOVA followed by Duncan’s multiple range test (SPSS 13.0, SPSS Inc., Chicago, IL, USA). The differences were considered significant at *p* < 0.05.
